# Systematic Analyses of the Role of the Reader Protein of *N*^6^-Methyladenosine RNA Methylation, YTH Domain Family 2, in Liver Hepatocellular Carcinoma

**DOI:** 10.3389/fmolb.2020.577460

**Published:** 2020-12-02

**Authors:** Xiang-yang Shao, Jin Dong, Han Zhang, Ying-song Wu, Lei Zheng

**Affiliations:** ^1^Department of Laboratory Medicine, Nanfang Hospital, Southern Medical University, Guangzhou, China; ^2^Department of Nephrology, Renmin Hospital of Wuhan University (Eastern Hospital), Wuhan, China; ^3^School of Laboratory Medicine and Biotechnology, Institute of Antibody Engineering, Southern Medical University, Guangzhou, China

**Keywords:** YTHDF2, liver hepatocellular carcinoma, *N*^6^-methyladenosine (m6A) RNA methylation, prognosis, tumor-immune infiltration

## Abstract

**Background:**

YTH domain family (YTHDF) 2 acts as a “reader” protein for RNA methylation, which is important in tumor regulation. However, the effect of YTHDF2 in liver hepatocellular carcinoma (LIHC) has yet to be elucidated.

**Methods:**

We explored the role of YTHDF2 in LIHC based on publicly available datasets [The Cancer Genome Atlas (TCGA), International Cancer Genome Consortium (ICGC), and Gene Expression Omnibus (GEO)]. A bioinformatics approach was employed to analyze *YTHDF2*. Logistic regression analyses were applied to analyze the correlation between YTHDF2 expression and clinical characteristics. To evaluate the effect of YTHDF2 on the prognosis of LIHC patients, we used Kaplan–Meier (K–M) curves. Gene set enrichment analysis (GSEA) was undertaken using TCGA dataset. Univariate and multivariate Cox analyses were used to ascertain the correlations between YTHDF2 expression and clinicopathologic characteristics with survival. Genes co-expressed with YTHDF2 were identified and detected using publicly available datasets [LinkedOmics, University of California, Santa Cruz (UCSC), Gene Expression Profiling Interactive Analysis (GEPIA), and GEO]. Correlations between YTHDF2 and infiltration of immune cells were investigated by Tumor Immune Estimation Resource (TIMER) and GEPIA.

**Results:**

mRNA and protein expression of YTHDF2 was significantly higher in LIHC tissues than in non-cancerous tissues. High YTHDF2 expression in LIHC was associated with poor prognostic clinical factors (high stage, grade, and T classification). K–M analyses indicated that high YTHDF2 expression was correlated with an unfavorable prognosis. Univariate and multivariate Cox analyses revealed that YTHDF2 was an independent factor for a poor prognosis in LIHC patients. GSEA revealed that the high-expression phenotype of YTHDF2 was consistent with the molecular pathways implicated in LIHC carcinogenesis. Analyses of receiver operating characteristic curves showed that YTHDF2 might have a diagnostic value in LIHC patients. YTHDF2 expression was associated positively with SF3A3 expression, which implied that they may cooperate in LIHC progression. YTHDF2 expression was associated with infiltration of immune cells and their marker genes. YTHDF2 had the potential to regulate polarization of tumor-associated macrophages, induce T-cell exhaustion, and activate T-regulatory cells.

**Conclusion:**

YTHDF2 may be a promising biomarker for the diagnosis and prognosis of LIHC and may provide new directions and strategies for LIHC treatment.

## Introduction

Liver cancer is one of the deadliest types of cancer. About 90% of primary liver cancers are liver hepatocellular carcinoma (LIHC). The latter results in >700,000 deaths and 850,000 new cases annually globally and is the fifth most prevalent cancer type worldwide (Tomita et al., 2011; Torre et al., 2015; Llovet et al., 2016). LIHC remains a major challenge of treatment due to its high morbidity and mortality, lack of effective diagnosis and treatment, and extremely poor prognosis (Mittal and El-Serag, 2013).

Efficacious treatments for LIHC are surgery, chemoradiotherapy, neoadjuvant therapy, local ablation, liver transplantation, or immunotherapy. However, the treatment results are not good, and mortality rates are high (Hsiao et al., 2017; Lee et al., 2017; Shen et al., 2017). There is a lack of effective biomarkers to predict the prognosis. Thus, discovering reliable prognostic biomarkers for LIHC is very important.

The initiation and progression of LIHC are regulated by several factors (El-Serag, 2011; Laursen, 2014). *N*^6^-Methyladenosine (m^6^A) modification was identified first in the 1970s and is the predominant form of mRNA methylation in eukaryotes (Du et al., 2019). m^6^A modification plays an important part in mRNA splicing; translation of protein biosynthesis; export, localization, and stability of mRNA; virus infection; and stem-cell differentiation (Fu et al., 2014; Meyer and Jaffrey, 2014; Chandola et al., 2015; Mendel et al., 2018). Recent studies have shown that methylation of m^6^A RNA is related to the initiation and progression of diseases, embryonic development (Wang Y. et al., 2014), and cancer (Cui et al., 2017; Kwok et al., 2017; Deng et al., 2018). m^6^A methylation is regulated by a group of proteins, including methyltransferases [“writers” (METTL3, METTL14, and WTAP)], binding proteins {“readers” [YTH domain family (YTHDF) 1, YTHDF2, YTHDF3, YTHDC1, YTHDC2]}, and demethylases [“erasers” (ALKBH5 and FTO)] (Li et al., 2017; Yang et al., 2018).

The outcome of m^6^A-modified mRNAs is based on the YTH (YT521-B homology) domain family protein (Meyer and Jaffrey, 2017). Among them, YTHDF2 was the first m^6^A reader protein to be identified and studied most widely and affects mRNA stability (Wang X. et al., 2014). YTHDF2 combines specifically with m^6^A-containing mRNA, regulates the stability of target RNA, and participates in regulation of a series of physiologic or pathologic processes (Wang X. et al., 2014; Li L. J. et al., 2018). Some studies have reported that YTHDF2 has an important role in acute myeloid leukemia (Paris et al., 2019). Inhibition of YTHDF2 expression advances expansion of hematopoietic stem cells (Li Z. et al., 2018). In pancreatic cancer, knockdown of YTHDF2 expression inhibits cell proliferation and promotes the migration and invasion of cells (Chen et al., 2017).

Using various publicly available databases, we investigated YTHDF2 expression and its relationship with the prognosis of cancer patients. Also, the correlation between YTHDF2 expression and clinical characteristics was investigated, and gene set enrichment analysis (GSEA) and co-expression analyses were undertaken. Finally, the correlation between YTHDF2 and infiltration of immune cells was studied in the tumor environment *via* the Tumor Immune Estimation Resource (TIMER) database.

We used databases to analyze associations between *YTHDF2* expression and the clinical prognosis of patients with LIHC. In this way, we provided suggestions and evidence for further study of the underlying mechanism between YTHDF2 and the tumorigenesis and progression of LIHC. Our study may aid in the use of a promising biomarker for the diagnosis and treatment of LIHC.

## Materials and Methods

### Dataset Acquisition and Bioinformatics Analyses

RNA-seq transcriptome data and corresponding clinicopathologic information were obtained from publicly available datasets. Datasets showing LIHC expression (GSE14520, GSE63898, and GSE64041) were downloaded from the Gene Expression Omnibus (GEO)^1^ database. Datasets showing the profile of mRNA expression of normal liver tissue were obtained from the Genotype-Tissue Expression (GTEx)^2^ database. RNA-seq data from The Cancer Genome Atlas (TCGA)-LIHC and International Cancer Genome Consortium (ICGC)-LIRI-JP cohorts were obtained from TCGA^3^ database and ICGC^4^ database, respectively. In TCGA-LIHC and ICGC-LIRI-JP cohorts, data on level 3 expression (RNA-seq transcriptome data) of LIHC patients and clinical-feature data of patients were arranged and analyzed further (Supplementary Table 1). The clinical-feature data of patients were extracted retrospectively from medical records (age, sex, clinical stage, histology grade, and Tumor–Node–Metastasis (TMN) classification). All RNA-seq data underwent normalization. Analyses of protein expression in clinical specimens were done using the Human Protein Atlas (HPA)^5^ database. We analyzed YTHDF2 expression in different cancer types in the TIMER^6^ database. Analyses of YTHDF2 expression in TCGA-LICH and ICGC-LIRI-JP cohorts were done using box plots to visualize differences in expression for discrete variables.

### Analyses of the Association Between YTHDF2 Expression and Clinical Characteristics, and Survival Analyses

Logistic regression analyses and the Wilcoxon signed-rank test were applied to identify the correlation between YTHDF2 expression and the clinical characteristics of LIHC. Correlation between variables of clinicopathologic characteristics and overall survival (OS) was determined by univariate and multivariate Cox regression analyses. Disease-free survival (DFS) and OS in LIHC patients with high and low expression of YTHDF2 were compared by Kaplan–Meier analyses with log-rank tests using the survival and survminer package within R (R Project for Statistical Computing, Vienna, Austria). To reduce the “noise” of disease-irrelevant deaths, in the ICGC-LIRI-JP cohort, survival duration >3 years was truncated to “3 years,” and the status of the corresponding patient was set to “alive.” In TCGA-LIHC cohort, survival duration >5 years was truncated to 5 years, and the status of the corresponding patient was set to “alive.”

### Gene Set Enrichment Analysis

GSEA is a computational method that determines whether a set of genes defined *a priori* shows significant concordant differences between two biological states (groups of high and low YTHDF2 expression in the present study) (Mootha et al., 2003; Subramanian et al., 2005). In the present study, a Hallmark gene set (h.all.v6.2.symbols.gmt), Biocarta gene set (c2.cp.biocarta.v6.2.symbols.gmt), and Kyoto Encyclopedia of Genes and Genomes (KEGG) gene set (c2.cp.kegg.v6.2.symbols.gmt) were applied to explore the potential mechanisms underlying YTHDF2 expression in LIHC, and GSEA was used to normalize data using the GSEA-3.0 tool (Liberzon et al., 2011). In general, gene sets with a false discovery rate (FDR) <0.25, absolute value of the normalized enrichment score (NES) ≥ 1.0, and normalized *P* < 0.05 were considered to be enriched significantly.

### Co-expression Genes Analysis

#### Analyses of Co-expressed Genes

The LinkedOmics database^7^ was used to screen genes that were co-expressed with YTHDF2 (absolute value of Pearson’s correlation coefficient > 0.4) and they were shown as heatmaps or volcano plots (Vasaikar et al., 2018). We used “clusterProfiler” within R package to undertake functional annotations for co-expressed genes. The *P*-adjust and *q*-value had to be < 0.05 to be considered significant categories in terms used in the Gene Ontology (GO) database and KEGG database. To evaluate the potential protein–protein interaction (PPI) of co-expressed genes, a PPI network was constructed using the Search Tool for the Retrieval of Interacting Genes/Proteins (STRING) database^8^. PPI pairs were extracted with a minimum required interaction score of 0.4, and the PPI network was visualized *via* Cytoscape 3.7.1^9^. The Reactome FI plugin within Cytoscape was employed to analyze networks of gene interactions. The top 10 core genes of the gene interaction network and PPI network were identified by the CytoHubba plugin within Cytoscape according to the degree score of each gene node. The Gene Expression Profiling Interactive Analysis (GEPIA) database^10^ was used to analyze the correlation between YTHDF2 and SF3A3, and we verified this correlation in other publicly available datasets [GEO; University of California, Santa Cruz (UCSC) Xena^11^ ].

#### Infiltration of Immune Cells

TIMER is a comprehensive resource for systematic analyses of infiltration of immune cells across diverse cancer types and includes 10,897 samples across 32 cancer types from TCGA. We analyzed the relationship between YTHDF2 expression and infiltration of immune cells [B cells, cluster of differentiation (CD)4+ T cells, CD8+ T cells, macrophages, neutrophils, and dendritic cells (DCs)] in LIHC using the “gene” module of TIMER, as well as the tumor purity. Furthermore, correlations between LAYN expression and marker genes of different immune cells were analyzed *via* correlation modules.

### Statistical Analyses

The Wilcoxon signed-rank test was applied to assess differences in expression of YTHDF2 mRNA between the LIHC group and normal group. Logistic regression analyses were done to evaluate the association between YTHDF2 expression and the clinical characteristics of patients with LIHC. The relationship between clinicopathologic variables and OS was analyzed by Cox regression analyses. In logistic regression, we classified high and low groups according to the median expression of YTHDF2 and SF3A3. In Cox regression analyses, expression of YTHDF2 and expression of SF3A3 were used as continuous variables. To evaluate the diagnostic efficacy of YTHDF2 expression, we drew the receiver operating characteristic (ROC) curve *via* the pROC package. The correlation between relative gene expression was undertaken by linear regression and Pearson’s correlation analyses. Data analyses were completed by R 3.6.0 and ActivePerl^®^ 5.26. *P* < 0.05 was considered significant.

## Results

### Clinical Data of Patients

Clinical-feature data and mRNA-expression data of LIHC patients were captured from TCGA and ICGC databases on June 2019 (Supplementary Table 1). In the TCGA-LIHC cohort, the clinical data of 348 LIHC patients were extracted retrospectively from medical records and comprised median age at the diagnosis (61 years), clinical stage (I = 173, II = 84, III = 86, and IV = 5), histology grade (G1 = 45, G2 = 171, G3 = 119, and G4 = 13), pathology classification for M (M0 = 267, M1 = 4, and MX = 77), pathology classification for T (T1 = 175, T2 = 86, T3 = 77, and T4 = 10), and pathology classification for N (N0 = 256, N1 = 4, and NX = 88). In the ICGC-LIRI-JP cohort, only the median age at the diagnosis (69 years), clinical stage (I = 40, II = 117, III = 80, and IV = 23), survival duration, and survival status of 260 LIHC patients were recorded.

### High YTHDF2 Expression in Liver Hepatocellular Carcinoma

**FIGURE 1:**
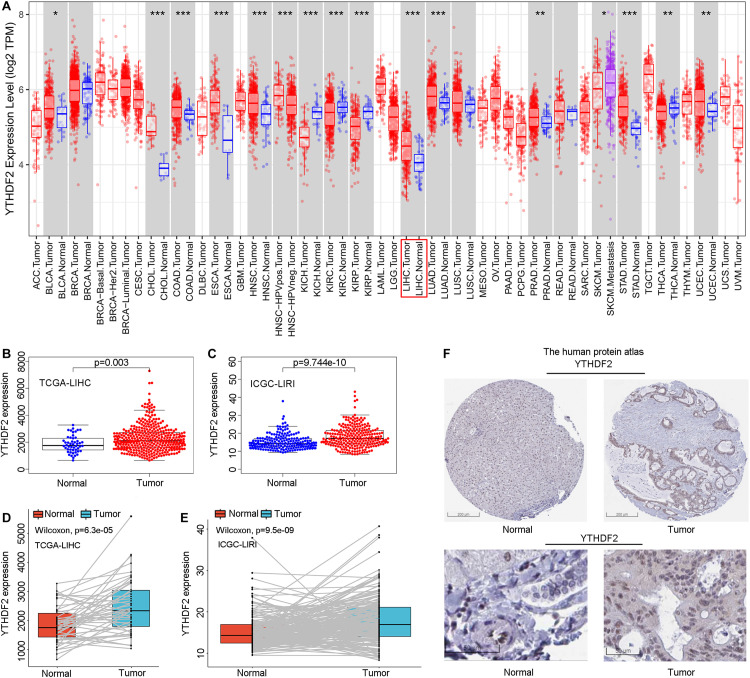
Pooled analyses of YTH domain family (YTHDF) 2 expression in liver hepatocellular carcinoma (LIHC). **(A)** YTHDF2 expression in different cancer types via the Tumor Immune Estimation Resource (TIMER) database. **(B)** YTHDF2 expression in 374 LIHC patients and 50 normal samples [The Cancer Genome Atlas (TCGA)-LIHC]. **(C)** YTHDF2 expression in 243 LIHC patients and 202 normal samples [International Cancer Genome Consortium (ICGC)-LIRI]. **(D)** YTHDF2 expression in 50 paired LIHC tissues and corresponding adjacent non-tumor tissues (TCGA-LIHC). **(E)** YTHDF2 expression in 199 paired LIHC tissues and corresponding adjacent non-tumor tissues (ICGC-LIRI). *P < 0.05, **P < 0.01, ***P < 0.001. **(F)** Expression of YTHDF2 protein in LIHC tissues and normal liver tissues (HPA). Scale bars, 200 and 50 μm.

Initially, we used an online tool (TIMER) to investigate YTHDF2 expression in different cancer types. In multiple cancer types (Figure 1A), YTHDF2 expression in cancer tissues was significantly higher than that in adjacent normal tissues. These cancer types were bladder urothelial carcinoma, cholangiocarcinoma, colon adenocarcinoma, esophageal carcinoma, head and neck cancer, chromophobe renal cell carcinoma, renal clear cell carcinoma, renal papillary cell carcinoma, LIHC, lung adenocarcinoma, adenocarcinoma of the prostate gland, gastric adenocarcinoma, carcinoma of the thyroid gland, and uterine corpus endometrial carcinoma. We focused mainly on the role of YTHDF2 in LIHC. To demonstrate the relationship between YTHDF2 expression and clinical relevance, we further analyzed YTHDF2 expression in publicly available datasets [HPA, TCGA-LIHC cohort, and ICGC-LIRI-JP (validation cohort)] (Figure 1). LIHC patients (*n* = 374), normal samples (*n* = 50), and 50 LIHC tissues and corresponding adjacent non-tumor tissues were included in TCGA-LIHC cohort. The ICGC-LIRI-JP cohort comprised 260 LIHC patients, 202 normal samples, and 199 LIHC tissues, and the corresponding adjacent non-tumor tissues were included in TCGA-LIHC cohort. YTHDF2 expression was significantly higher in LIHC tissues than that in non-tumor tissues (*P* < 0.05) (Figures 1B,C) or paired non-tumor tissues (*P* < 0.05) (Figures 1D,E). In the HPA, we analyzed expression of YTHDF2 protein. We discovered that compared with YTHDF2 staining in normal liver tissue, YTHDF2 staining was stronger in LIHC tissue (Figure 1F). Next, in other exposed datasets (GEO and GTEx), we assessed YTHDF2 expression in LIHC tissues and normal tissues (Table 1). The results were consistent with those in TCGA-LIHC and ICGC-LIRI cohorts. These data showed that YTHDF2 expression was upregulated in LIHC.

**TABLE 1:** The expression level of YTHDF2 in public LIHC datasets.

**Dataset**	***P*-value**	**Type**	**Nums**	**Mean**	***SD***
TCGA + GTEx	4.671e-09	Tumor	374	1766.39	448.72
		Normal	160	1539.58	412.6
GSE14520-GPL3921	1.863e-14	Tumor	225	7.52	0.55
		Normal	220	7.16	0.50
GSE63898-GPL13667	3.366e-05	Tumor	228	8.64	0.423
		Normal	168	8.48	0.356
GSE64041-GPL6244	4.16-04	Tumor	60	8.48	0.16
		Normal	60	8.35	0.16
GSE14520-GPL571	0.005	Tumor	22	7.58	0.61
		Normal	21	7.24	0.28

*SD, Standard Deviation.*

### High Expression of YTHDF2 Is Associated With Clinical Characteristics

**FIGURE 2:**
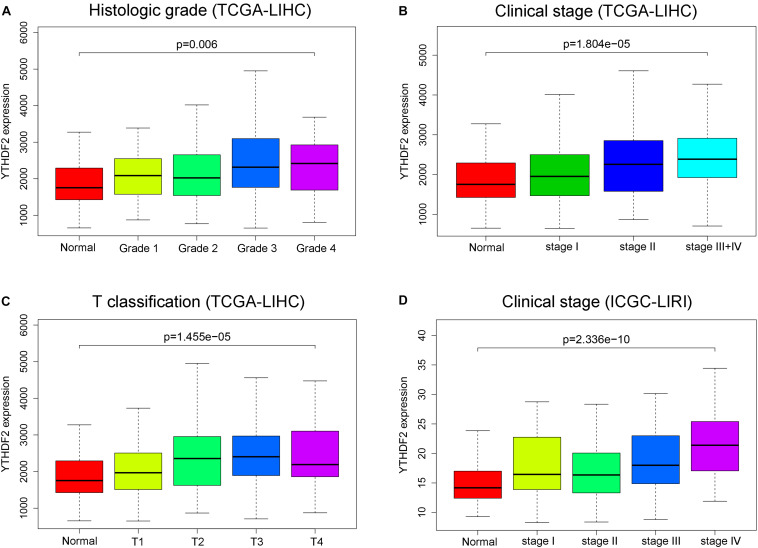
YTH domain family (YTHDF) 2 is overexpressed and associated positively with poor prognostic clinical factors in liver hepatocellular carcinoma (LIHC) patients. **(A)** YTHDF2 expression in normal individuals or LIHC patients with grade 1, 2, 3, or 4 tumors [The Cancer Genome Atlas (TCGA)-LIHC]. **(B)** YTHDF2 expression in normal individuals or in LIHC patients with stage 1, 2, or 3 + 4 (TCGA-LIHC). **(C)** YTHDF2 expression in normal individuals or in LIHC patients with T classification 1, 2, 3, or 4 (TCGA-LIHC). **(D)** YTHDF2 expression in normal individuals or in LIHC patients with stage 1, 2, 3, or 4 [International Cancer Genome Consortium (ICGC)-LIRI].

There were a total of 375 and 232 LIHC patients with YTHDF2 expression and clinical information in TCGA-LIHC and ICGC-LIRI, respectively. Then, we evaluated the association between YTHDF2 expression and the clinical characteristics of LIHC patients. Univariate logistic regression analyses showed that YTHDF2 expression was correlated substantially with poor prognostic clinical factors (grade, T classification, and stage) in LIHC patients (Table 2). In TCGA-LIHC-cohort, high expression of YTHDF2 was connected significantly with high clinical stage [odds ratio (OR) = 2.56 for III–IV vs. I; 2.16 for II vs. I], histology grade (1.66 for poor vs. good), and T classification (2.51 for T3–T4 vs. T1) (*P* < 0.05 for all). In the ICGC-LIRI cohort, a high clinical stage (IV vs. I) was correlated significantly with high YTHDF2 expression. High expression of YTHDF2 was related significantly to T classification, stage, and histology grade (*P* < 0.05 for all) (Figures 2A–D). These results demonstrated that in LIHC patients, the high-expression group of YTHDF2 tended to progress to a more advanced grade and stage than the low-expression group of YTHDF2.

**TABLE 2:** Relationship between clinical features and YTHDF2 expression in LIHC patients (logistic regression).

**Clinical characteristics**	**TCGA-LIHC**			**ICGC-LIRI-JP**		
	**Total (*N*)**	**OR (95%CI)**	***p*-value**	**Total (*N*)**	**OR (95%CI)**	***p*-value**
Age (continuous)	370	0.99 (0.98–1.01)	0.23	232	0.99 (0.97–1.02)	0.68
Gender (female vs. male)	371	0.88 (0.57–1.36)	0.56	231	2.38 (1.31–4.43)	**0.01**
Grade (poor vs. well)	365	1.66 (1.09–2.57)	**0.03**	NA		
T classification (T3-T4 vs. T1)	274	2.51 (1.51–4.22)	**0.01**	NA		
M classification (M1 vs. M0)	270	0.33 (0.02–2.60)	0.34	NA		
Stage (II vs. I)	257	2.16 (1.28–3.67)	**0.00**	142	1 (0.47–2.14)	1
Stage (III–IV vs. I)	261	2.56 (1.52–4.35)	**0.00**	126	1.26 (0.86–1.88)	0.24
Stage (IV vs. I)	NA			55	1.50 (1.03–2.27)	**0.04**
Stage (III + IV vs. I + II)	261	1.26 (0.86–1.88)	0.24	232	1.67 (0.98–2.85)	0.06

*OR, Odds ratio; CI, confidence interval. Bold values indicate p < 0.05. p < 0.05 was considered statistically significant.*

### YTHDF2 Is an Independent Predictor of Poor Survival (Disease-Free Survival and Overall Survival) in Liver Hepatocellular Carcinoma

**FIGURE 3:**
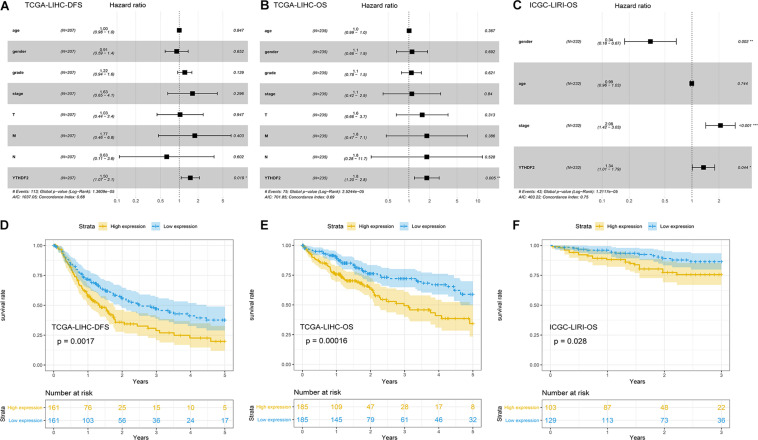
Potential prognostic importance of high YTH domain family (YTHDF) expression in patients with liver hepatocellular carcinoma (LIHC). **(A)** Forest plot of the association between risk factors and disease-free survival (DFS) in The Cancer Genome Atlas (TCGA)-LIHC patients. **(B)** Forest plot of the association between risk factors and overall survival (OS) in TCGA-LIHC patients. **(C)** Forest plot of the association between risk factors and OS in International Cancer Genome Consortium (ICGC)-LIRI patients. **(D,E)** Kaplan–Meier survival curve of YTHDF expression in OS and DFS in LIHC (TCGA-LIHC). **(F)** Kaplan–Meier survival analysis of YTHDF expression in OS in LIHC (ICGC-LIRI).

Univariate Cox analyses indicated that the prognosis was related to the clinical stage, T classification, M classification, and YTHDF2 expression (Table 3). High expression of YTHDF2 was correlated significantly with a poor prognosis [TCGA-LIHC cohort: OS, hazard ratio (HR) = 2.05, 95% confidence interval (CI): 1.39–3.04, *P* < 0.001; DFS, 1.72, 1.23–2.40, *P* < 0.001; ICGC-LIRI cohort: OS, 1.06, 1.01–1.11, *P* < 0.001]. Multivariate analyses demonstrated that YTHDF2 expression was an independent predictor for survival in LIHC patients (TCGA-LIHC cohort: OS, HR = 1.80, 95% CI: 1.20–2.80; *P* < 0.05; DFS, 1.50, 1.07–2.10, *P* < 0.05. ICGC-LIRI cohort: OS, 1.34, 1.01–1.79, *P* < 0.05) (Figures 3A–C). OS and DFS of high- and low-expression groups of YTHDF2 were compared by Kaplan–Meier analyses with log-rank tests in LIHC patients. Kaplan–Meier curves revealed that LIHC patients with high YTHDF2 expression were correlated with a poor prognosis (*P* < 0.05) (Figures 3D–F).

**TABLE 3:** Cox regression analysis the association between clinicopathological variables and overall survival.

**Parameter**	**TCGA-LIHC Univariate analysis**				**ICGC-LIRI Univariate analysis**	
	**OS**		**DFS**		**OS**	
	**HR(95%CI)**	***p*-value**	**HR(95%CI)**	***p*-value**	**HR(95%CI)**	***p*-value**
Age	1.01 (0.99–1.02)	0.59	1.00 (0.98–10.1)	0.59	1.00 (0.97–1.03)	0.90
Gender	0.78 (0.49–1.25)	0.30	0.72 (0.48–1.06)	0.09	0.52(0.28–0.97)	0.04
Grade	1.02 (0.75–1.39)	0.91	1.15 (0.89–1.47)	0.28	NA	
Stage	1.86 (1.46–2.39)	**0.00**	1.73 (1.41–2.13)	**1.40e-07**	2.15(1.49–3.11)	**4.13e-05**
T classification	1.80 (1.43–2.27)	**0.00**	1.67 (1.37–2.02)	**2.27e-07**	NA	
M classification	3.85 (1.21–12.28)	**0.02**	4.56 (1.43–14.57)	**0.01**	NA	
N classification	2.02 (0.49–8.28)	0.33	1.34 (0.33–5.44)	0.68	NA	
YTHDF2	2.05 (1.39–3.04)	**0.00**	1.72 (1.23–2.40)	**0.00**	1.46 (1.11–1.93)	**0.00**

*OS, overall survival; DFS, disease-free survival; HR, hazard ratio; NA, not available. Bold values indicate p < 0.05. p < 0.05 was considered statistically significant.*

### Functional Annotation of YTHDF1

#### Gene Set Enrichment Analysis and Potential Function of YTHDF2 Expression

We wished to investigate the potential function of YTHDF2 expression in LIHC. We undertook GSEA and used the MSigDB Collection (c2.cp.biocarta.v6.2.symbols.gmt, h.all.v6.2.symbols.gmt, c2.cp.kegg.v6.2.symbols.gmt) to examine the enriched gene sets at different expression of YTHDF2. We selected the most significantly enriched signaling pathways based on the NES (Supplementary Table 2). GSEA suggested that mitotic spindle, protein secretion, integrin pathway, Wnt pathway, meiosis of oocytes, regulation of autophagy, spliceosome, and vascular endothelial growth factor, as well as expression of mammalian target of rapamycin (mTOR), phosphoinositide 3-kinase/protein kinase B/mTOR, and p53 pathways were enriched differentially in the YTHDF2 high-expression group. These results were consistent with the molecular pathways implicated in LIHC carcinogenesis (Couri and Pillai, 2019). Due to the limited space, only six pathways of high expression are listed in Supplementary Figure 1, but all indicated the potential role of YTHDF2 in LIHC development.

#### Co-expression of YTHDF2 mRNA in Liver Hepatocellular Carcinoma

**FIGURE 4:**
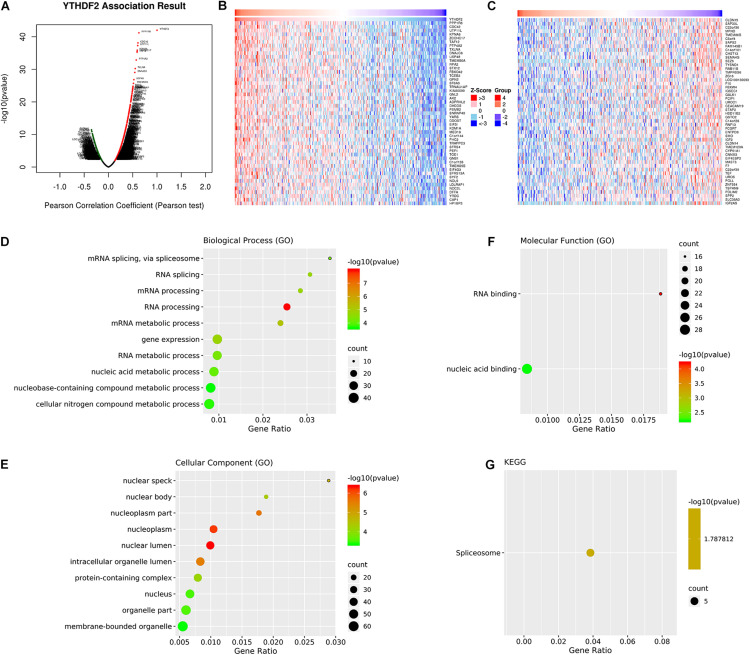
Analyses of genes co-expressed with YTH domain family (YTHDF) 2. **(A)** Volcano plot of all the genes co-expressed with YTHDF2 identified by Pearson’s test. The top 50 co-expressed genes positively **(B)** and negatively **(C)** associated with YTHDF2 in liver hepatocellular carcinoma (LIHC). Gene Ontology (GO) annotations and Kyoto Encyclopedia of Genes and Genomes (KEGG) pathway analyses of genes co-expressed genes with YTHDF2. **(D)** Biological processes. **(E)** Cellular components. **(F)** Molecular functions. **(G)** Pathway analyses using the KEGG database.

We wished to investigate further the underlying regulation of YTHDF2 expression in LICH. Thus, we analyzed the co-expression profiles with YTHDF2 in LIHC using the LinkedOmics database. Seventy-eight genes were co-expressed significantly with YTHDF2 mRNA (absolute value of Pearson’s correlation ≥ 0.4, FDR > 0.05) (Supplementary Table 3). The volcano plot (Figure 4A) demonstrated that the global genes were correlated with YTHDF2 as identified by Pearson’s test. Heatmaps (Figures 4B,C) revealed the top 50 genes negatively and positively associated with YTHDF2 in LIHC. Analyses using the GO database showed that the genes co-expressed with YTHDF2 were located mainly in nuclear speckles, nuclear body, nucleoplasm, and nuclear lumen (Figure 4E), where they participated primarily in binding of nucleic acids and RNA (Figure 4F), thereby suggesting effects on RNA processing, RNA splicing, and metabolic processes (Figure 4D). Pathway analyses using the KEGG database revealed that these genes were enriched mainly in spliceosome pathways (Figure 4G).

#### Construction of a Network of YTHDF2 Co-expressed Genes and Identification of Potential “Hub” Genes

**FIGURE 5:**
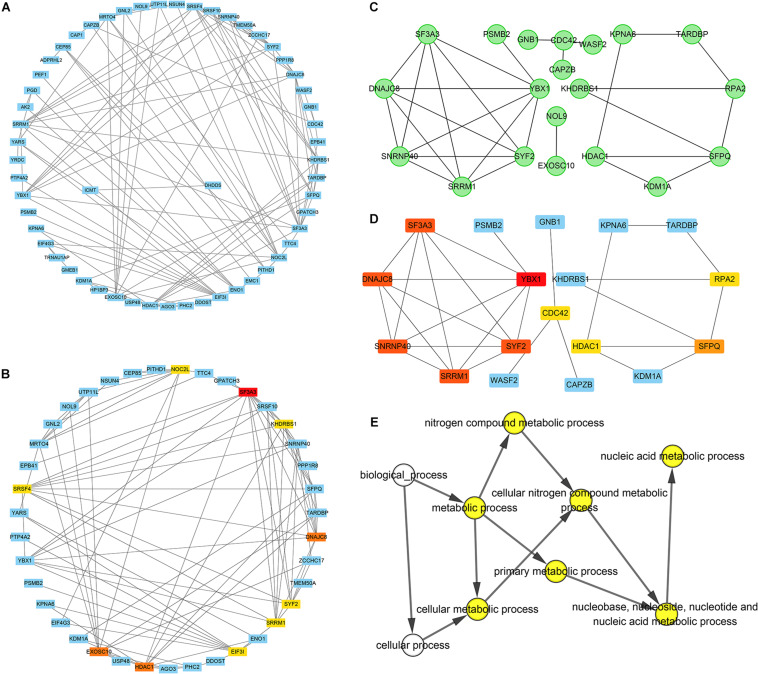
Construction of an interaction network with YTH domain family (YTHDF) 2 positive-correlated genes and identification of potential hub genes. **(A)** Protein–protein interaction (PPI) network of YTHDF1 co-expressed genes. **(B)** The top 10 hub genes identified in the PPI network. **(C)** Gene network of YTHDF2 co-expressed genes. **(D)** The top 10 hub genes identified in the gene interaction network. **(E)** Analyses of biological processes of five duplicate hub genes. *P* < 0.05 was considered significant.

A PPI network of 78 co-expressed genes was constructed using the STRING database and visualized *via* Cytoscape 3.7.1 (Figure 5A). We used the Reactome FI plugin within Cytoscape for analyses of gene interaction networks (Figure 5B). The top 10 core genes (Table 4 and Figures 5C,D) were identified by the CytoHubba plugin within Cytoscape according to the degree score of each gene node. We explored the biological processes of five duplicate hub genes using the BINGO plugin of Cytoscape. These hub genes participated mainly in the cellular metabolic processes of nitrogen compounds and nucleic acid metabolism, suggesting that they may affect protein biosynthesis in cells (Figure 5E). Among these core genes, the highest degree score was for *SF3A3*, so it may be the most important key core gene.

**TABLE 4:** Top ten potential hub genes were identified in PPI and Gene network.

**Gene symbol**	**Gene description**	**PPI network Degree**	**Gene network Degree**
SF3A3	Splicing factor 3a subunit 3	13	5
HDAC1	Histone deacetylase 1	10	3
DNAJC8	DnaJ heat shock protein family (Hsp40) member C8	10	5
SRRM1	Serine and arginine repetitive matrix 1	9	5
SYF2	SYF2 pre-mRNA splicing factor	9	5
EIF3I	Eukaryotic translation initiation factor 3 subunit I	9	NA
NOC2L	NOC2 like nucleolar associated transcriptional repressor	9	NA
SRSF4	Serine and arginine rich splicing factor 4	9	NA
KHDRBS1	KH RNA binding domain containing, signal transduction associated 1	9	NA
EXOSC10	Exosome component 10	10	NA
YBX1	Y-box binding protein 1	NA	6
SNRNP40	Small nuclear ribonucleoprotein U5 subunit 40	NA	5
SFPQ	Splicing factor proline and glutamine rich	NA	4
CDC42	Cell division cycle 42	NA	3
RPA2	Replication protein A2	NA	3

#### Co-expression of YTHDF2 and SF3A3

**FIGURE 6:**
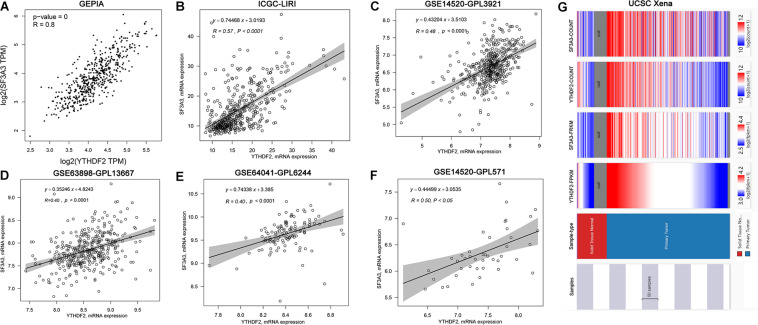
Correlation analyses of YTH domain family (YTHDF) 2 expression and SF3A3 expression in publicly available datasets. **(A)** Gene Expression Profiling Interactive Analysis (GEPIA). **(B)** International Cancer Genome Consortium (ICGC)-LIRI. **(C)** GSE14520-GPL3921. **(D)** GSE63898-GPL13667. **(E)** GSE64041-GPL6244. **(F)** GSE14520-GPL571. **(G)** UCSC Xena.

Correlation analyses using the GEPIA database showed that expression of YTHDF2 and SF3A3 had a high correlation coefficient (*R* = 0.8, *P* < 0.05) (Figure 6A). In other publicly available datasets (GEO and cBioPortal), a positive correlation between YTHDF2 and SF3A3 transcripts was validated (Figures 6B–F). Finally, we confirmed this phenomenon further in the UCSC Xena database (Figure 6G). These results suggested that YTHDF2 expression and SF3A3 expression had a strong positive correlation, suggesting that they may be “functional partners” in LIHC.

#### SF3A3 Expression and Prognostic Value in Liver Hepatocellular Carcinoma

**FIGURE 7:**
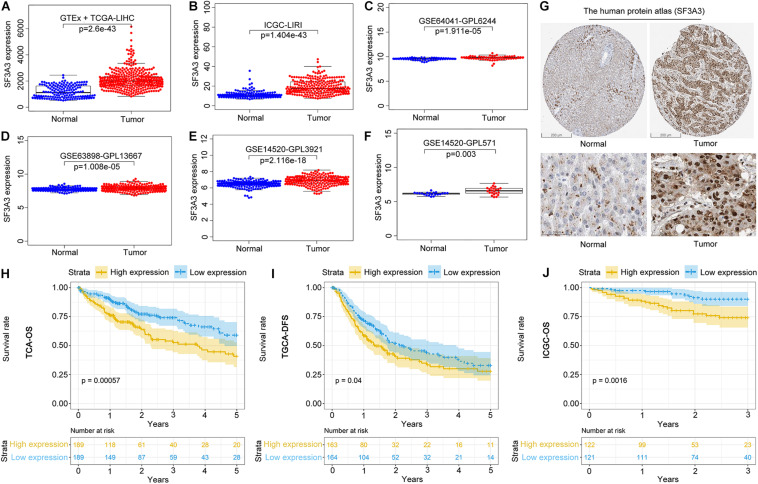
Pooled analyses of the expression profile of SF3A3 were investigated using publicly available data sets in liver hepatocellular carcinoma (LIHC). SF3A3 expression in publicly available data sets. **(A)** GTEx + The Cancer Genome Atlas (TCGA)-LIHC. **(B)** International Cancer Genome Consortium (ICGC)-LIRI. **(C)** GSE64041-GPL6244. **(D)** GSE63898-GPL13667. **(E)** GSE14520-GPL3921. **(F)** GSE14520-GPL571. **(G)** Expression of SF3A3 protein in LIHC tissues and normal liver tissues (HPA). Scale bars, 200 and 50 μm. Kaplan–Meier survival analyses showing SF3A3 expression as overall survival (OS) **(H)** and disease-free survival (DFS) **(I)** in LIHC (TCGA-LIHC). **(J)** Kaplan–Meier survival analyses showing SF3A3 expression as OS in LIHC (ICGC-LIRI).

We wished to analyze gene expression of SF3A3 in LIHC, so we used publicly available datasets. Dataset analyses indicated that SF3A3 expression was significantly higher in LIHC tissues than in non-tumor tissues (Figures 7A–F). In the HPA, we analyzed expression of SF3A3 protein: compared with SF3A3 staining in normal liver tissue, SF3A3 staining was stronger in LIHC tissue (Figure 7G). Subsequently, we investigated the prognostic value of SF3A3 in TCGA-LIHC and ICGC-LIRI-JP cohorts, and we confirmed that high expression of SF3A3 was associated significantly with a decrease in OS and DFS in LIHC (Figures 7H–J).

#### Diagnostic Value of High YTHDF2 Expression in Liver Hepatocellular Carcinoma

**FIGURE 8:**
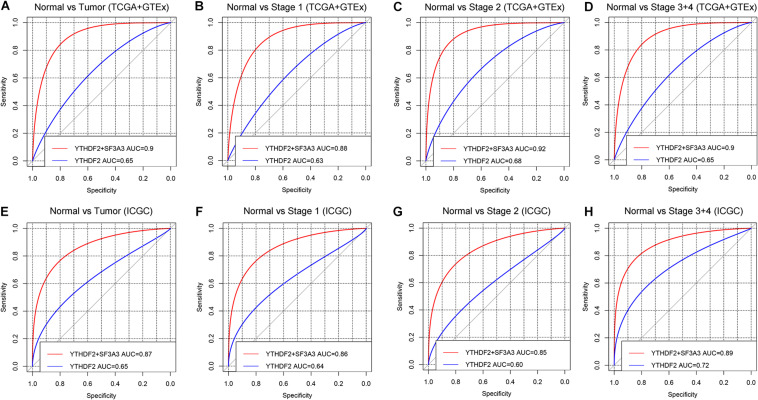
Diagnostic value of high expression of YTH domain family (YTHDF) 2 and SF3A3 in liver hepatocellular carcinoma (LIHC). **(A,E)** Receiver operating characteristic (ROC) curve for expression of YTHDF2 and SF3A3 in normal tissue and tumor tissue. **(B–D,F–H)** Subgroup analyses for ROC curves for stages I, II, and III + IV.

ROC curves were used to assess the diagnostic value of YTHDF2 in LIHC patients and healthy individuals from GTEx + TCGA-LIHC and ICGC-LIRI datasets. In the normal group vs. all tumor groups, the area under the ROC curve (AUC) of YTHDF2 was 0.65 in the GTEx + TCGA-LIHC, and ICGC-LIRI cohorts (Figures 8A,E). Furthermore, we assessed the diagnostic value of YTHDF2 in a subgroup of LIHC patients with regard to clinical stage. Analyses of ROC curves demonstrated that high YTHDF2 expression might have a diagnostic value for LIHC patients of different clinical stages: the AUC was 0.63 and 0.64 for stage I, 0.68 and 0.60 for stage II, and 0.65 and 0.72 for stage III + IV in the TCGA and ICGC cohorts, respectively (Figure 8). Combined diagnosis of YTHD2 and SF3A3 could improve the diagnostic efficiency significantly, with all AUC values > 0.85 (Figure 8). These results suggested that expression of YTHDF2 and SF3A3 might have a diagnostic value for LIHC patients.

#### YTHDF2 Expression Is Correlated With Infiltration of Immune Cells in Liver Hepatocellular Carcinoma

**FIGURE 9:**
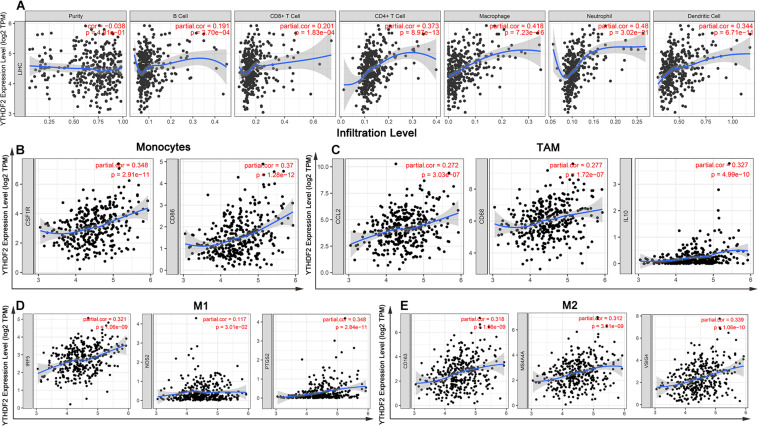
YTH domain family (YTHDF) 2 expression has significant correlations with infiltration of immune cells and macrophage polarization in liver hepatocellular carcinoma (LIHC). **(A)** YTHDF2 expression was significantly positively related to infiltration of B cells, CD8 + T cells, CD4 + T cells, macrophages, neutrophils, and dendritic cells in LIHC. **(B–D)** Scatterplots of correlations between YTHDF2 expression and gene markers of monocytes **(B)**, TAMs **(C)**, M1 macrophages **(D)**, and M2 macrophages **(E)** in LIHC. Monocyte markers: CD86 and CSF1R. TAM markers: CCL2, CD68, and IL10. M1 macrophages markers: NOS2, IRF5, and PTGS2. M2 macrophages markers: CD163, VSIG4, and MS4A4A.

Tumor-infiltrating lymphocytes are independent prognostic elements of survival and can reflect metastasis to sentinel lymph nodes (Ohtani, 2007; Azimi et al., 2012). To further broaden the understanding of YTHDF2, we investigated if YTHDF2 expression was associated with the degree of infiltration of immune cells in LIHC using the TIMER database. YTHDF2 expression had a significant correlation with various “immune signatures,” which included infiltration levels of B cells (*r* = 0.19, *P* = 3.70 × 10^–4^), CD8+ T cells (0.20, 1.83 × 10^–4^), CD4 + T cells (0.37, 8.97 × 10^–13^), macrophages (0.42, 7.23 × 10^–16^), neutrophils (0.48, 3.02 × 10^–21^), and DCs (0.34, 6.71 × 10^–4^) in LIHC (Figure 9A). These results strongly indicated that YTHDF2 expression was a crucial participant in infiltration of immune cells in LIHC, particularly macrophages and neutrophils.

#### Correlation Analyses Between Immune Marker Genes and YTHDF2 Expression

Immune cells can be activated in different states (Fridlender et al., 2009; Murray, 2017). We focused on the relationship between YTHDF2 and marker genes of various types of immune cells [CD8+ T cells, monocytes, T cells (general), M1 and M2 macrophages, B cells, neutrophils, tumor-associated macrophages (TAMs), natural killer cells, AND DCs] in LIHC in GEPIA and TIMER databases. We also analyzed T cells with different functions, such as T-helper type 1 (Th1), Th17, Th2, T-regulatory (Treg), T follicular helper, and exhausted T cells (Table 5). After tumor purity-correlated adjustments, we found a significant correlation between YTHDF2 expression and most marker genes of various types of immune cells and different functional T cells in LIHC. YTHDF2 expression was strongly correlated with expression of monocytes, TAMs, DCs, and M1 and M2 macrophages (Table 5). Expression of C–C motif chemokine ligand-2, interleukin (IL) 10 and CD68 of TAMs, interferon regulatory factor-5, and cyclo-oxygenase (COX) 2 of the M1 phenotype, as well as MS4A4A, VSIG4, and CD163 of the M2 phenotype was correlated significantly with YTHDF2 expression in LIHC (*P* < 0.0001) (Figures 9B-E). In addition, we assessed expression of YTHDF2 and the immune markers stated above in GEPIA and ICGC-LIRI databases. The correlation of YTHDF2 expression with marker genes of monocytes and TAMs was consistent with that in the TIMER database (Table 6). These findings suggested that YTHDF2 may regulate macrophage polarization in LIHC.

**TABLE 5:** The relevance analysis of YTHDF2 and relate genes of immune cells in TIMER dataset.

**Description**	**Gene markers**	**LIHC**			
		**None**		**Purity**	
		**Cor**	***P***	**Cor**	***P***
CD8+ T cell	CD8A	0.17	**1.38e-03**	0.16	**2.79e-03**
	CD8B	0.1	6.51e-02	0.09	1.04e-01
T cell (general)	CD3D	0.1	6.85e-02	0.1	7.71e-02
	CD3E	0.1	6.98e-02	0.1	9.61e-02
	CD2	0.1	8.35e-02	0.1	9.61e-02
B cell	CD19	0.16	**1.64e-03**	0.14	**7.33e-03**
	CD79A	0.06	2.33e-01	0.05	3.36e-01
Monocyte	CD86	0.34	**3.02e-11**	0.37	**1.28e-11**
	CD115 (CSF1R)	0.32	**3.78e-10**	0.35	**2.91e-11**
TAM	CCL2	0.26	**6.55e-07**	0.27	**3.03e-07**
	CD68	0.27	**1.73e-07**	0.28	**1.72e-07**
	IL10	0.31	**1.15e-09**	0.33	**4.99e-10**
M1 Macrophage	INOS (NOS2)	0.12	**2.19e-02**	0.12	**3.01e-02**
	IRF5	0.33	**6.96e-11**	0.32	**1.06e-09**
	COX2(PTGS2)	0.32	**5.72e-10**	0.35	**2.84e-11**
M2 Macrophage	CD163	0.30	**6.23e-09**	0.32	**1.56e-09**
	VSIG4	0.31	**9.23e-10**	0.34	**1.06e-10**
	MS4A4A	0.28	**3.02e-08**	0.31	**3.31e-09**
Neutrophils	CD66b(CEACAM8)	0.09	9.71e-02	0.09	8.68e-02
	CD11b (ITGAM)	0.38	**3.93e-14**	0.4	**1.15e-14**
	CCR7	0.13	**1.22e-02**	0.12	**2.63e-02**
Natural killer cell	KIR2DL1	0.07	1.75e-01	0.05	3.83e-01
	KIR2DL3	0.17	**8.81e-04**	0.18	**1.11e-03**
	KIR2DL4	0.18	**5.39e-04**	0.17	**2.03e-03**
	KIR3DL1	0.13	**1.01e-02**	0.14	**8.82e-03**
	KIR3DL2	0.07	2.03e-01	0.07	1.75e-01
	KIR3DL3	0.06	2.08e-01	0.04	4.44e-01
	KIR2DS4	0.14	**8.15e-03**	0.14	**9.35e-03**
Dendritic cell	HLA-DPB1	0.21	**4.14e-05**	0.21	**9.95e-05**
	HLA-DQB1	0.13	**1.45e-02**	0.12	**2.86e-02**
	HLA-DRA	0.28	**5.06e-08**	0.29	**4.58e-08**
	HLA-DPA1	0.27	**1.45e-07**	0.28	**1.42e-07**
	BDCA-1(CD1C)	0.13	**1.35e-02**	0.12	**3.00e-02**
	BDCA-4(NRP1)	0.53	**0.00**	0.53	**6.53e-26**
	CD11c (ITGAX)	0.32	**2.09e-10**	0.35	**2.02e-11**
Th1	T-bet (TBX21)	0.13	**1.46e-02**	0.13	**1.98e-02**
	STAT4	0.16	**2.36e-03**	0.16	**2.36e-03**
	STAT1	0.35	**4.8e-12**	0.34	**9.26e-11**
	IFN-γ (IFNG)	0.17	**9.83e-04**	0.18	**1.11e-03**
	TNF-α (TNF)	0.30	**8.67e-09**	0.32	**1.76e-09**
Th2	GATA3	0.21	**4.74e-05**	0.23	**1.14e-05**
	STAT6	0.40	**2.63e-15**	0.38	**4.39e-13**
	STAT5A	0.39	**3.75e-15**	0.39	**6.03e-14**
	IL13	0.11	**3.28e-02**	0.10	**6.58e-02**
Tfh	BCL6	0.48	**0.00**	0.49	**7.11e-22**
	IL21	0.12	**2.57e-02**	0.11	**3.59e-02**
Th17	STAT3	0.49	**0.00**	0.50	**1.54e-23**
	IL17A	0.07	2e-01	0.09	1.11e-01
Treg	FOXP3	0.26	**5.27e-07**	0.26	**6.63e-07**
	CCR8	0.39	**5.24e-15**	0.42	**4.56e-16**
	STAT5B	0.46	**0.00**	0.46	**9.2e-20**
	TGFβ (TGFB1)	0.27	**1.13e-07**	0.29	**4.76e-08**
T cell exhaustion	PD-1 (PDCD1)	0.15	**4.13e-03**	0.13	**1.39e-02**
	CTLA4	0.14	**8.47e-03**	0.12	**1.18e-02**
	LAG3	0.09	7.31e-02	0.08	1.36e-01
	TIM-3 (HAVCR2)	0.35	**3.14e-12**	0.40	**2.51e-14**
	GZMB	0.06	2.18e-01	0.03	5.46e-01

*None, correlation without adjustment. Purity, tumor purity-correlated adjustments. TAM, Tumor-associated macrophages; Cor, R-value of Spearman’s correlation. Bold font: *p* value < 0.05.*

**TABLE 6:** Correlation analysis of YTHDF2 expression and markers genes of monocyte and macrophages in ICGC-LIRI and GEPIA databases.

**Description**	**Gene markers**	**GEPIA**		**ICGC-LIRI**	
		**Tumor**		**Tumor**	
		
		***R***	***p*-value**	***R***	***p*-value**
Monocyte	CD86	0.35	**4.4e-12**	0.24	**0.0001**
	CD115 (CSF1R)	0.52	**2.3e-27**	0.17	**0.0070**
TAM	CCL2	0.26	**4.4e-07**	0.23	**0.0002**
	CD68	0.29	**1.3e-08**	0.15	**0.018**
	IL10	0.32	**2.1e-10**	0.17	**0.008**
M1 Macrophage	INOS (NOS2)	0.16	**0.0021**	0.05	0.467
	IRF5	0.32	**5.2e-10**	0.27	**1.581e-05**
	COX2 (PTGS2)	0.34	**1.1e-11**	0.17	**0.007**
M2 Macrophage	CD163	0.18	**0.00062**	0.007	0.90
	VSIG4	0.31	**8.5e-10**	0.13	**0.042**
	MS4A4A	0.29	**1.1e-08**	0.12	0.05

*Bold font: *p* value < 0.05.*

High expression of YTHDF was associated with high infiltration of DCs. Expression of DC marker genes (e.g., HLA-DRA, HLA-DRA1, BDCA-4, and CD11c) was correlated significantly with YTHDF2 expression. Furthermore, for Tregs, expression of FOXPM3 and TGFB1 was correlated positively with YTHDF2 expression in LIHC. DCs promote tumor metastasis by increasing the number of Tregs and inhibiting the cytotoxicity of CD8 + T cells (Sawant et al., 2012). Whether YTHDF2 is a driving factor mediating DCs and tumor metastases merits further study.

We also observed that YTHDF2 expression had a significant correlation with the marker genes of Tregs and T-cell exhaustion (e.g., CCR8, FOXP3, STAT5B, and TIM-3) (Table 5). FOXP3 can inhibit cytotoxic T cells attacking cancer cells. *TIM-3* is a key gene that regulates T-cell exhaustion and has a high positive correlation with YTHDF2 expression, indicating that high expression of YTHDF2 has a crucial role in TIM-3-mediated T-cell exhaustion. Hence, these results further demonstrate that YTHDF2 expression was correlated significantly with infiltration of immune cells in LIHC. Hence, in the tumor microenvironment, YTHDF2 has an important role in immune escape. YTHDF2 may also affect the occurrence and development of LIHC by regulating the infiltration of immune cells of different phenotypes.

## Discussion

RNA methylation is the major modification of RNA. m^6^A is a predominant internal mRNA modification (Li and Mason, 2014; Roundtree et al., 2017). Studies have shown that methylation of m^6^A RNA is related to the initiation and progression of diseases, including cancer. YTHDF2 is a m^6^A reader. As a key enzyme participating in the regulation of RNA methylation, it can combine specifically with m^6^A-containing mRNA and can regulate the stability of target RNA (Wang X. et al., 2014; Li L. J. et al., 2018). The reader proteins of m^6^A methylation are YTHDF1, YTHDF2, YTHDF3, YTHDC1, and YTHDC2. We found that only YTHDF1 and YTHDF2 were related to the survival of LIHC patients. The prognostic role of YTHDF1 in LIHC has been reported (Zhao et al., 2018). Studies on the potential prognostic role of YTHDF2 in LIHC are lacking: the present study was conducted to fill this knowledge gap. We comprehensively analyzed the expression and role of YTHDF2 in a large cohort of LIHC patients. We employed bioinformatics to analyze R-seq data on TCGA-LIHC (training group) and ICGC-LIRI-JP (validation group) cohorts. We demonstrated, for the first time, that high expression of YTHDF2 in LIHC was correlated significantly with clinicopathologic variables (high clinical stage, histology grade, and T classification) and a poor prognosis.

Differences in expression of YTHDF2 mRNA between the LIHC group and normal group were analyzed in multiple LIHC datasets. Then, Kaplan–Meier survival analyses, univariate and multivariate Cox regression analyses, and ROC curve analyses were used to ascertain the value of YTHDF2. Logistic regression analyses were applied to identify the relationship between YTHDF2 expression and clinical characteristics. In addition, GSEA, as well as analyses of co-expression, and infiltration of immune cells were done to study how YTHDF2 participates in the evolution and progression of LIHC.

Studies have shown that knockdown of YTHDF2 expression inhibits cell proliferation and promotes the migration and invasion of pancreatic cancer cells (Chen et al., 2017). Zhong et al. (2019) showed that YTHDF2 can inhibit the proliferation and growth of cells by destroying the stability of epidermal growth factor receptor mRNA in LIHC. Chen et al. (2018) demonstrated that RNA m^6^A-METTL3 promotes LIHC progression *via* YTHDF2-dependent post-transcriptional silencing of SOCS2 expression. The conclusions of the two studies mentioned above are inconsistent, which suggests the dual role of YTHDF2 in tumor cells.

Here, we revealed that YTHDF2 expression was significantly higher in LIHC than in normal liver tissue and was correlated significantly with clinicopathologic variables (high clinical stage, histology grade, and T classification). Analyses of Kaplan–Meier survival curves showed that high YTHDF2 expression was associated with a poor prognosis (OS and DFS) in LIHC patients. Univariate and multivariate Cox regression analyses demonstrated that YTHDF2 was an independent factor for a poor prognosis in LIHC patients. Hence, YTHDF2 may be a valuable prognostic biomarker for patients with LIHC. The GSEA results of the high-expression phenotype of YTHDF2 are consistent with the molecular pathways implicated in LIHC pathogenesis. ROC curve analyses showed that YTHDF2 might have a diagnostic value in LIHC patients. Taken together, our data suggest the potential role of YTHDF2 in LIHC development.

Co-expression analyses demonstrated that YTHDF2 expression was associated positively with SF3A3 expression. SF3A3 is obtained from purified spliceosomes and is an important component of the SF3A RNA splicing complex (Chiara et al., 1994). SF3A3 participates in the replication and repair of DNA and transcriptional regulation (Yang et al., 2012). SF3A3 can also inhibit the activity of the tumor-suppressor gene p53. Silencing SF3A3 expression can increase p53 expression, thereby inducing the cell-cycle arrest and death of tumor cells (Siebring-van Olst et al., 2017). In addition, the interaction between SF3A3 and CSR1 is essential for cell death (Zuo et al., 2017). Therefore, we analyzed SF3A3 expression in LIHC tissues and non-tumor tissues: SF3A3 expression in LIHC tissues was significantly higher than that in non-tumor tissues. Survival results showed that high expression of SF3A3 was associated with a poor prognosis in LIHC. We speculate that YTHF2 and SF3A3 may participate jointly in the development and progression of LIHC.

Another important aspect of this research was the correlation between YTHDE2 expression and immune signatures in LIHC. YTHDF2 expression was associated with infiltration of immune cells and marker genes of immune cells (Tables 5, 6). The correlation between YTHDF2 expression and markers of M2 macrophages was slightly stronger than that of markers of M1 macrophages and was related to TAM markers, which suggested the potential regulatory role of YTHDF2 in TAM polarization. Furthermore, YTHDF2 expression was correlated positively with markers of Tregs (TGFB1, CCR8, FOXP3, and STAT5B), which indicated that YTHDF2 could activate Tregs. YTHDF2 expression was also related to *TIM-3* (a key gene for T-cell exhaustion) expression. Thus, we speculate that YTHDF2 has the potency to induce T-cell exhaustion. Taken together, these results show that YTHDF2 has an important role in the recruitment and regulation of infiltrating immune cells in LIHC.

More in-depth experiments and clinical trials are needed to verify the value of YTHDF2 in LIHC. One limitation of our study was that we did not consider potential confounders for the association between YTHDF2 expression and survival (e.g., type of radiotherapy/chemotherapy and location of patient enrollment).

## Conclusion

High expression of YTHDF2 was associated with a poor prognosis and, together with increased infiltration of immune cells, could be an independent prognostic indicator for patients with LIHC. GSEA results were consistent with the molecular pathways implicated in LIHC pathogenesis in a high-expression phenotype of YTHDF2. Co-expression analyses confirmed the upregulation of expression of YTHDF2 and SF3A3 in LIHC, both of which were associated with a poor prognosis of LIHC. In addition, YTHDF2 was involved in regulation of TAMs and T_regs_. Thus, YTHDF2 may have an important role in infiltration of immune cells and help to improve immunomodulatory therapy in LIHC patients.

We believe that YTHDF2 may be a promising biomarker for the diagnosis and prognosis of LIHC patients and may provide new directions and strategies for their treatment. However, the specific regulatory mechanisms merit further exploration.

## Data Availability Statement

The raw data supporting the conclusions of this article will be made available by the authors, without undue reservation.

## Ethics Statement

Ethical review and approval was not required for the study on human participants in accordance with the local legislation and institutional requirements. Written informed consent for participation was not required for this study in accordance with the national legislation and the institutional requirements.

## Author Contributions

XS and LZ: conceptualization. XS and JD: methodology, formal analysis, and visualization. XS and HZ: investigation. XS, LZ, JD, HZ, and YW: resources. XS: writing—original draft preparation. XS, HZ, and JD: writing—review and editing. LZ and YW: supervision. LZ: funding acquisition. All authors have read and agreed to the published version of the manuscript.

## Conflict of Interest

The authors declare that the research was conducted in the absence of any commercial or financial relationships that could be construed as a potential conflict of interest.

## Footnotes

1 www.ncbi.nlm.nih.gov/geo/

2 https://gtexportal.org/home/

3 https://tcga-data.nci.nih.gov/tcga/

4 https://dcc.icgc.org/

5 http://www.proteinatlas.org/

6 https://cistrome.shinyapps.io/timer/

7 http://www.linkedomics.org/login.php/

8 http://string-db.org/

9 https://cytoscape.org/

10 http://gepia.cancer-pku.cn/

11 https://xena.ucsc.edu/

## Supplementary Material

Supplementary Figure 1 The top-six most significant gene set enrichment analysis (GSEA) results. **(A)** mitotic spindle, **(B)** protein secretion, **(C)** integrin_pathway, **(D)** wnt_pathway, **(E)** oocyte_meiosis, **(F)** regulation_of_autophagy. NES, normalized enrichment score; NOM *p*-val, normalized *P*-value.

Supplementary Table 1 Clinical features of LIHC patients in TCGA and ICGC.

Supplementary Table 2 Enrichment plots from gene set enrichment analysis (GSEA) in MSigDB collection c2.cp.kegg.v6.2.symbols.gmt).

Supplementary Table 3 YTHDF2 co-expression genes in HCC (LinkedOmics).
