# Supplementary figures and images for: Systematic Analyses of the Role of the Reader Protein of N6-Methyladenosine RNA Methylation, YTH Domain Family 2, in Liver Hepatocellular Carcinoma
Source: Front Mol Biosci. 2020 Dec 2;7:577460. doi: 10.3389/fmolb.2020.577460 (PMC7738478; doi:10.3389/fmolb.2020.577460)

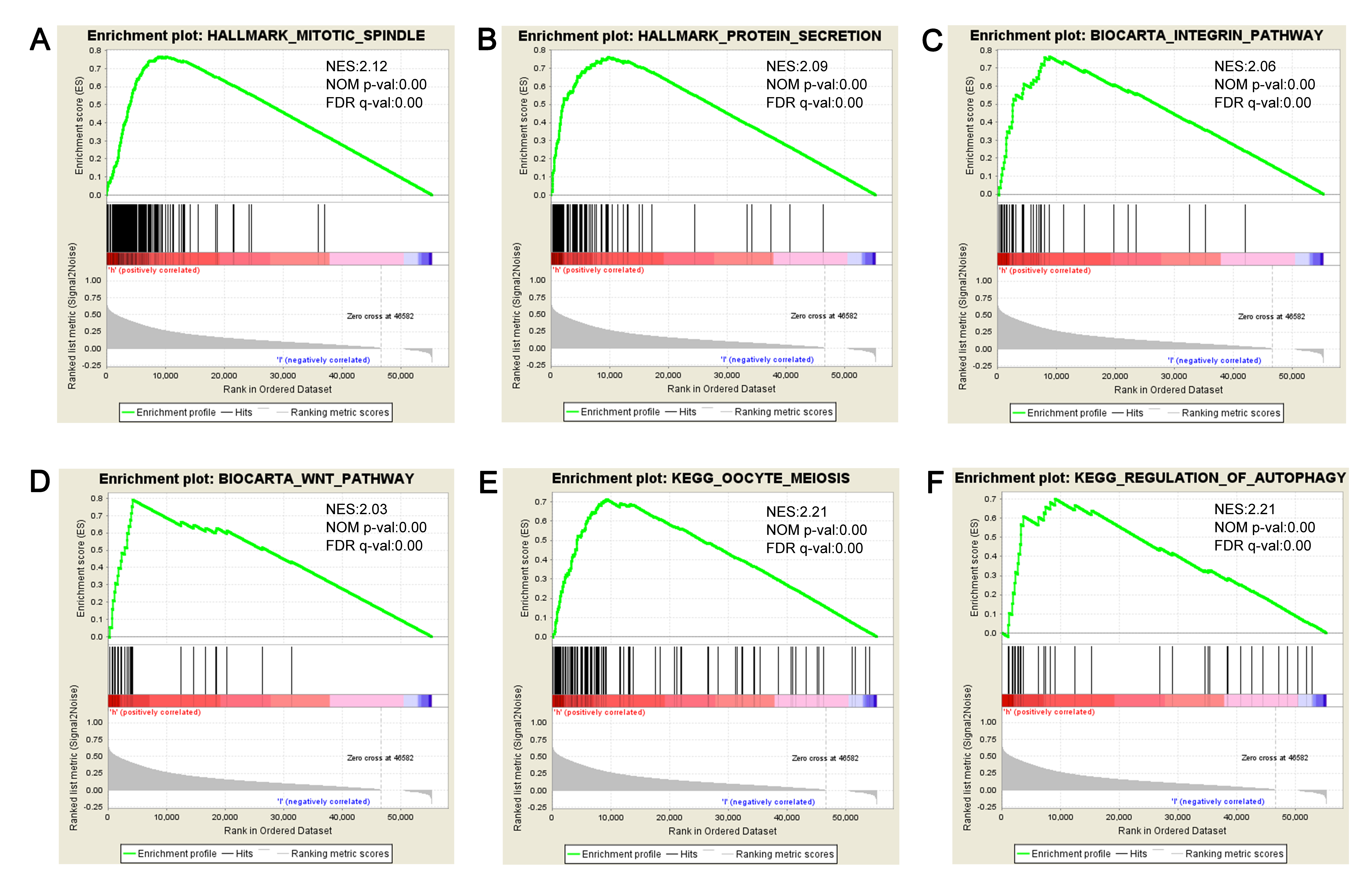

Supplement: Supplementary Figure 1 — The top-six most significant gene set enrichment analysis (GSEA) results. (A) mitotic spindle, (B) protein secretion, (C) integrin_pathway, (D) wnt_pathway, (E) oocyte_meiosis, (F) regulation_of_autophagy. NES, normalized enrichment score; NOM p-val, normalized P-value. [file Image_1.tif]
