# Supplementary material for: Systematic Analyses of the Role of the Reader Protein of N6-Methyladenosine RNA Methylation, YTH Domain Family 2, in Liver Hepatocellular Carcinoma
Source: Front Mol Biosci. 2020 Dec 2;7:577460. doi: 10.3389/fmolb.2020.577460 (PMC7738478; doi:10.3389/fmolb.2020.577460)
Supplement: Supplementary Table 1 — Clinical features of LIHC patients in TCGA and ICGC. [file Table_1.docx]

**Supplementary Table 1. Clinical features of LIHC patients in TCGA and ICGC.**

| **Clinical features** | | **TCGA-LIHC** | |  | **ICGC-LIRI-JP** | |
| --- | --- | --- | --- | --- | --- | --- |
|  |  | **Total(348)** | **%** |  | **Total(260)** | **%** |
| **Age** |  | 61 (16-90) |  |  | 69 (31-89) |  |
| **Gender** | Female | 110 | 31.6 |  | 68 | 26.2 |
|  | Male | 238 | 68.4 |  | 192 | 73.8 |
| **Histologic grade** | G1 | 45 | 12.9 |  | NA | |
|  | G2 | 171 | 49.1 |  |  |  |
|  | G3 | 119 | 34.2 |  |  |  |
|  | G4 | 13 | 3.7 |  |  |  |
| **Stage** | I | 173 | 49.7 |  | 40 | 15.4 |
|  | II | 84 | 24.1 |  | 117 | 45 |
|  | III | 86 | 24.7 |  | 80 | 30.8 |
|  | IV | 5 | 1.4 |  | 23 | 8.8 |
| **T classification** | T1 | 175 | 50.3 |  | NA | |
|  | T2 | 86 | 24.7 |  |  |  |
|  | T3 | 77 | 22.1 |  |  |  |
|  | T4 | 10 | 2.9 |  |  |  |
| **M classification** | M0 | 267 | 76.7 |  | NA | |
|  | M1 | 4 | 1.15 |  |  |  |
|  | MX | 77 | 22.1 |  |  |  |
| **N classification** | N0 | 256 | 73.6 |  | NA | |
